# Morphology, Morphogenesis, and Molecular Phylogeny of a New Freshwater Ciliate, *Quadristicha subtropica* n. sp. (Ciliophora, Hypotrichia)

**DOI:** 10.3389/fmicb.2021.705826

**Published:** 2021-07-15

**Authors:** Chen Shao, Qi Gao, Alan Warren, Jingyi Wang

**Affiliations:** ^1^Laboratory of Protozoological Biodiversity and Evolution in Wetland, College of Life Sciences, Shaanxi Normal University, Xi’an, China; ^2^Department of Life Sciences, Natural History Museum, London, United Kingdom

**Keywords:** ciliates, morphology, new species, ontogenesis, phylogeny

## Abstract

The morphology and the regulation of cortical pattern associated with the cell size, division, and phylogenetic position of a new hypotrichous ciliate, *Quadristicha subtropica* n. sp. collected from a freshwater pond in southern China, were investigated. *Quadristicha subtropica* n. sp. is characterized as follows: size *in vivo* 60–115 μm × 25–45 μm; 19–21 adoral membranelles; buccal cirrus near anterior end of endoral and paroral; cirrus IV/3 at about level of buccal vertex; right marginal row begins ahead of buccal vertex; 11–16 right and 12–19 left marginal cirri; and dorsal cilia about 5 μm long. The basic morphogenetic process in *Q. subtropica* n. sp. is consistent with that of the type species, *Quadristicha setigera*. Phylogenetic analyses based on small subunit ribosomal DNA sequence data reveal that the systematic position of *Q. subtropica* n. sp. is rather unstable with low support values across the tree and the genus *Quadristicha* is not monophyletic.

## Introduction

Ciliates are one of the most species-rich groups within Protozoa and live in a variety of habitats, such as soil, freshwater, and seawater (Small and Lynn, 1985; Cheng et al., 2019; Bai et al., 2020; Lian et al., 2020; Wu et al., 2020; Zhao et al., 2020). Hypotrichia Stein, 1859 is considered to have the most complex morphology and morphogenesis within the phylum Ciliophora Doflein, 1901. They are thus increasingly recognized as being of significance to the study of cell biology, genetics, and ecology. Recent studies have revealed numerous new taxa of hypotrichs, suggesting that this group is even more diverse than previously supposed (Berger, 1999; Foissner, 2016; Song and Shao, 2017; Hu et al., 2019; Chen et al., 2020; Dong et al., 2020; Lu et al., 2020; Paiva, 2020; Park et al., 2020; Shao et al., 2020; Wang et al., 2020, 2021a; Xu et al., 2020; Zhang et al., 2020; Li et al., 2021a,b; Vďačný and Foissner, 2021).

Oxytrichidae Ehrenberg, 1830 is a species-rich family within the subclass Hypotrichia (Berger, 1999, 2018; Foissner, 2016). Recently, some new genera have been established for some of the *Oxytricha* species, namely, *Fragmospina* Foissner, 2016, *Paroxytricha* Foissner, 2016, *Monomicrocaryon* Foissner, 2016, *Quadristicha* Foissner, 2016, *Aponotohymena* Foissner, 2016 and *Oxytrichella* Foissner, 2016. *Quadristicha* Foissner, 2016 is an oxytrichid genus that is characterized by having a flexible body with 18 frontal-ventral-transverse cirri, two macronuclear nodules with a micronucleus in between, dorsal kineties that do not fragment during ontogenesis, and three caudal cirri. The type species is *Quadristicha setigera* (Stokes, 1891) Foissner, 2016.

In September 2018, an oxytrichid ciliate was isolated from a freshwater pond in Peninsula Lake Park, Wanning, Hainan Province, China. In the present study, we investigate its morphology, morphogenesis, and the phylogenetic position.

## Materials and Methods

*Quadristicha subtropica* n. sp. was isolated from a freshwater pond in Peninsula Lake Park in Wanning, China (18°41′03″N; 110°24′02″E), on September 12, 2018. Some bark and rotten leaves were taken together with water from the sampling site. Cells were cultured at the room temperature in the laboratory with mineral water (Nongfu Spring), enriched with rice. Although we failed to establish a clonal culture, no other oxytrichid morphotypes were present in the protargol preparations. Therefore, we are confident that the morphological, morphogenetic, and molecular studies reported here deal solely with the same species.

Cells were studied *in vivo* using a high-power oil immersion objective and differential interference contrast. Protargol (Wilbert, 1975) was used to reveal the infraciliature. Measurements of silvered specimens were performed with the imaging software cellSens Entry (Olympus). Drawings of live specimens are based on photographic records, and those of impregnated cells were made with a camera lucida. For clarity, parental cirri are shown only by outline, whereas new ones are shaded. Terminology follows Berger (1999) and Foissner (2016).

### DNA Extraction, PCR Amplification, and Sequencing

The genomic DNA extraction, PCR amplification, and gene sequencing were carried out according to Wang et al. (2021b).

### Phylogenetic Analyses

The SSU rDNA sequence of *Quadristicha subtropica* n. sp. was aligned with sequences of 70 other hypotrich species downloaded from GenBank database for phylogenetic analyses. Euplotid species were used as the outgroup taxa. Phylogenetic analyses were carried out according to Wang et al. (2021b).

## Results

### ZooBank Registration

Present work: urn:lsid:zoobank.org:pub:4CBB9B60-4158-4F2C-B603-02D50B4F8ABE *Quadristicha subtropica* n. sp.: urn:lsid:zoobank.org:act:A8FB0E8F-91F1-4768-8DEE-522580AA55B3.

### *Quadristicha subtropica* n. sp.

#### Diagnosis

**Figure 1:**
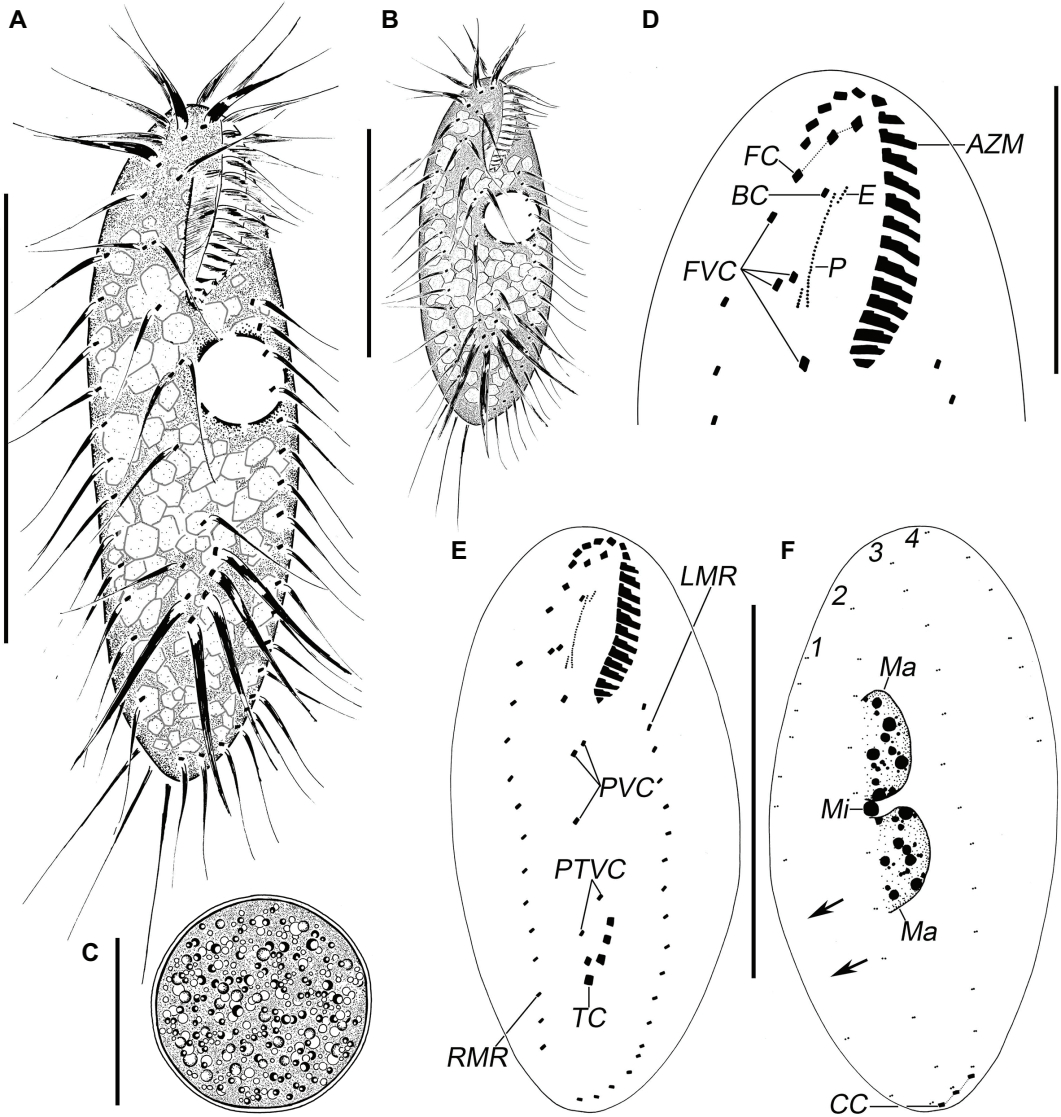
*Quadristicha subtropica* n. sp., morphology from life **(A–C)** and after protargol staining **(D–F)**. **(A,B)** Ventral views, to show different body shapes. **(C)** Resting cyst. **(D)** Detailed ventral view of the anterior region. **(E,F)** Ventral **(E)** and dorsal **(F)** view of the holotype specimen to demonstrate the infraciliature; arrows mark the gap in dorsal kinety 1. 1–4, dorsal kineties 1–4. Bars: 50 μm **(A,B)**; 30 μm **(C,D)**; 70 μm **(E,F)**.

**Figure 2:**
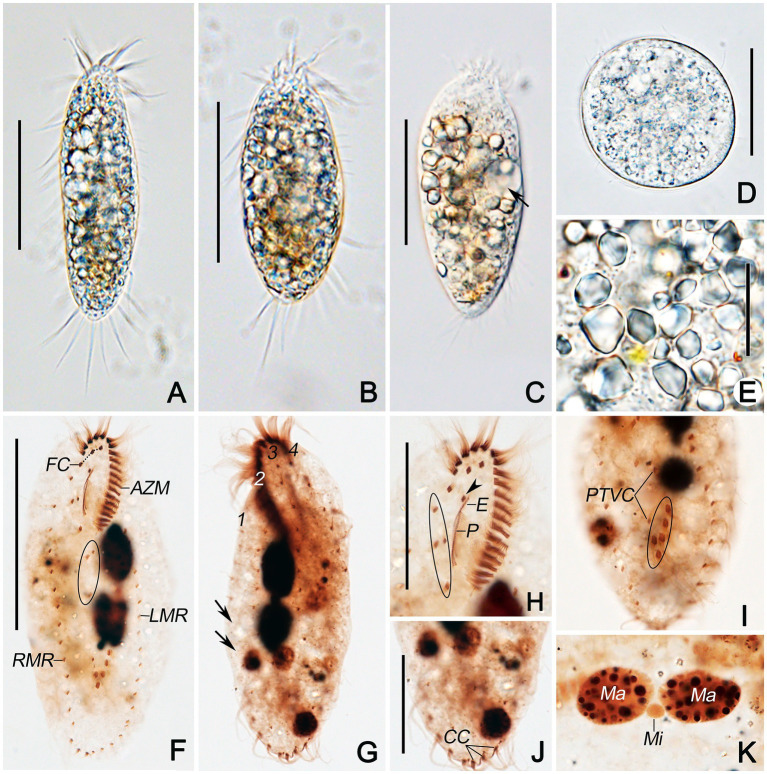
Photomicrographs of *Quadristicha subtropica* n. sp. from life **(A–E)** and after protargol staining **(F–K)**. **(A,B)** Ventral views of representative individuals to show different body shapes. **(C)** Ventral view, to denote contractile vacuole (arrow). **(D)** Resting cyst. **(E)** Crystals. **(F,H)** Ventral views to demonstrate the ciliature. Arrowhead marks the buccal cirrus; circle in **F** and **H** demonstrates the postoral ventral and the frontoventral cirri, respectively. **(I)** Ventral view of posterior end, to show transverse cirri (circle). **(G,J,K)** Dorsal views to demonstrate the dorsal kineties, caudal cirri, and nuclear apparatus. Arrows in **G** show the gap in dorsal kinety 1. 1–4, dorsal kineties 1–4. Bars: 50 μm **(A–C)**; 30 μm **(D,H,J)**; 20 μm **(E)**; 70 μm **(F)**.

**Figure 3:**
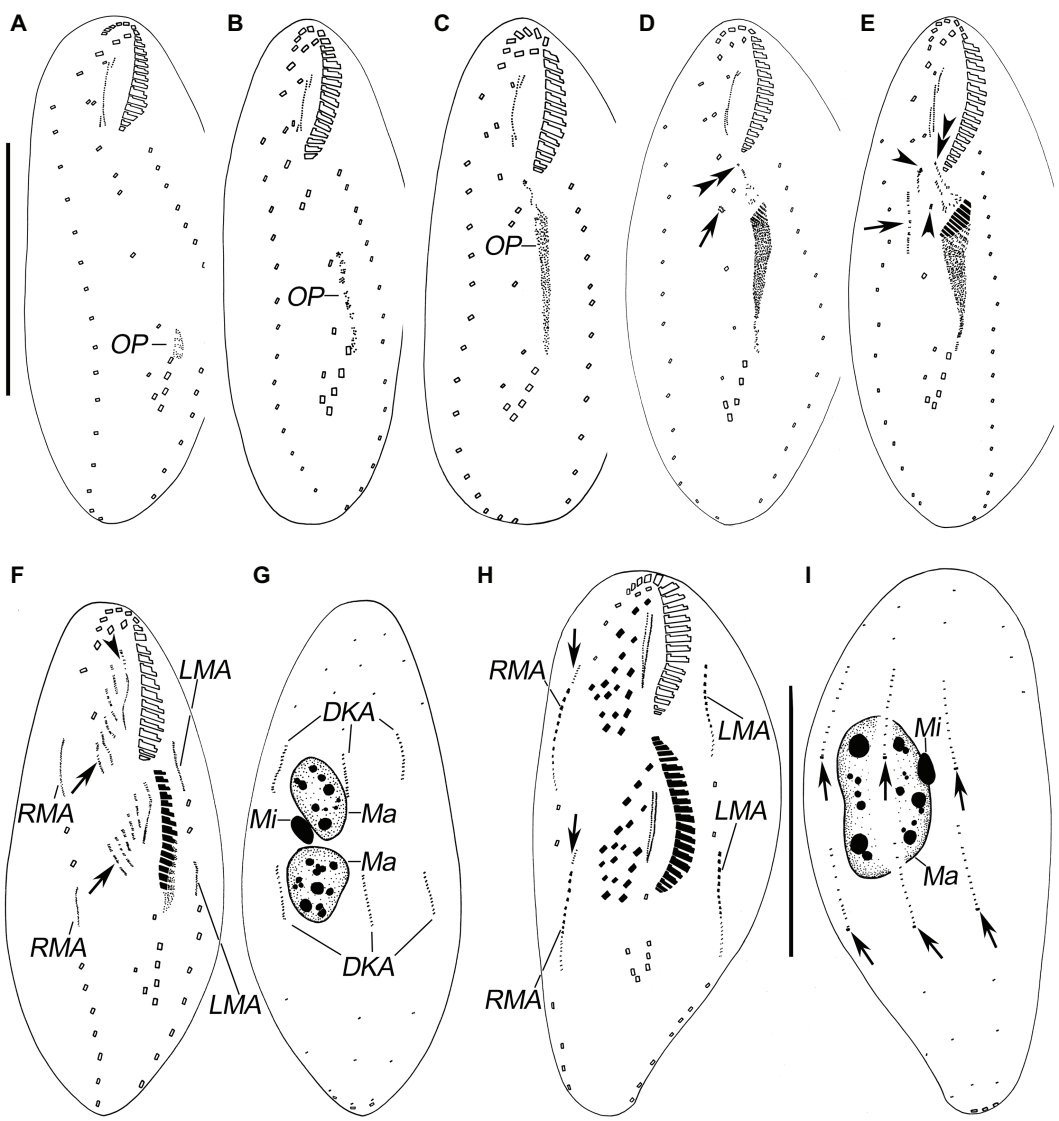
Morphogenesis of *Quadristicha subtropica* n. sp. after protargol staining. **(A–C)** Ventral views of very early dividers showing the newly formed oral primordium. **(D)** Ventral view of an early divider, arrow marks the dedifferentiation of cirrus V/4, and double arrowheads indicate the UM-anlage. **(E)** Ventral view of a slightly later divider, arrow shows the frontal-ventral-transverse cirral anlagen, arrowheads mark the dedifferentiation of cirri IV/2 and IV/3, and double arrowheads indicate the UM-anlage. **(F,G)** Ventral and dorsal view of a divider, arrows show frontal-ventral-transverse cirral anlagen and arrowhead denote the frontal cirrus separated from the frontal-ventral-transverse cirral anlage I. **(H,I)** Ventral and dorsal view of a mid-divider, arrows in **H** mark the dorsomarginal kineties anlagen and arrows in **I** demonstrate the newly formed caudal cirri. Bars: 50 μm **(A–G)**; 60 μm **(H,I)**.

**Figure 4:**
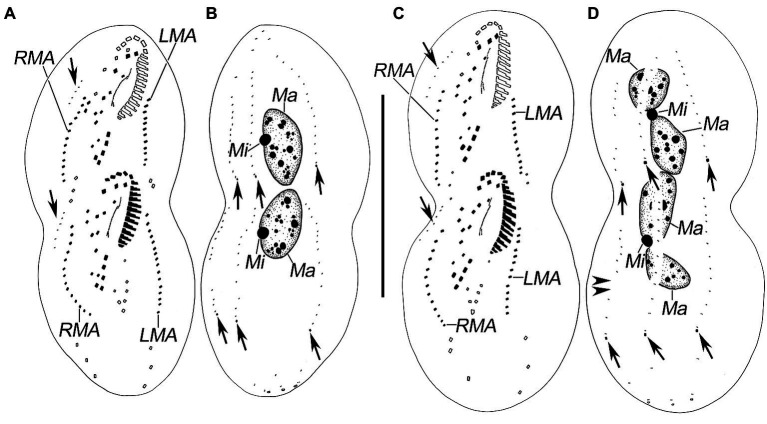
Late stages of morphogenesis in *Quadristicha subtropica* n. sp. after protargol staining. **(A,B)** Ventral and dorsal view of a late divider, to show the dorsomarginal kineties anlagen (arrows in **A**) and the caudal cirri (arrows in **B**). **(C,D)** Ventral and dorsal view of a very late divider to demonstrate the dorsomarginal kineties (arrows in **C**) and caudal cirri (arrows in **D**). Arrowheads in **D** show the gap in dorsal kinety 1. Bar: 70 μm.

**Figure 5:**
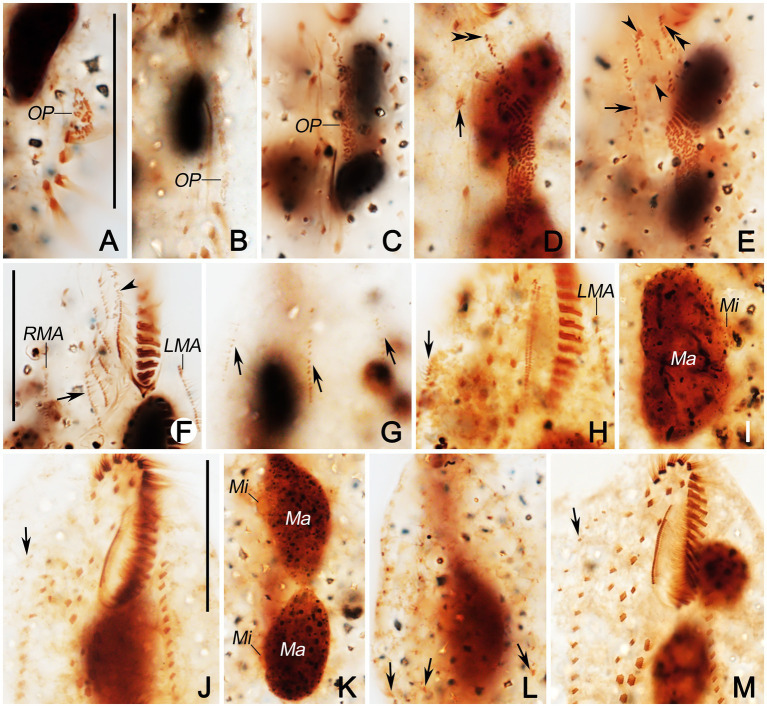
Photomicrographs of *Quadristicha subtropica* n. sp. during morphogenesis, after protargol staining. **(A–C)** Early morphogenetical stages in ventral views, showing the newly formed oral primordium. **(D)** Ventral view of early divider, arrow marks the dedifferentiation of cirrus V/4, and double arrowheads indicate the UM-anlage. **(E)** Ventral view of early divider, arrow shows the frontal-ventral-transverse cirral anlagen, arrowheads mark the dedifferentiation of cirri IV/2 and IV/3, and double arrowheads indicate the UM-anlage. **(F,G)** Ventral and dorsal view of a divider, arrow in **(F)** shows frontal-ventral-transverse cirral anlagen, arrowhead marks undulating membranelles anlage, and arrows in **(G)** show dorsal kineties anlagen. **(H)** Ventral view of a mid-divider, arrow marks the dorsomarginal kineties anlage. **(I)** Dorsal view, to denote the fusion of the macronuclear nodules. **(J,M)** Ventral views, to show the ciliature. Arrows mark the dorsomarginal kinety anlagen. **(K)** Dorsal views, to demonstrate the development of the macronuclear nodules. **(L)** Dorsal view, arrows point to the caudal cirri. Bars: 30 μm.

Size *in vivo* 60–115 μm × 25–45 μm, usually elliptical or elongate ovoid in shape. Cortical granules absent. 19–21 adoral membranelles (Figures 1A–F, 2A–K, 3A–I, 4A–D, 5A–M and Table 1). Buccal cirrus near anterior end of undulating membranes. Cirrus III/2 slightly ahead of level of cirrus VI/3. Cirrus IV/2 anterior to level of cirrus V/4. Distance between cirri V/2 and V/3 slightly longer than distance between cirri V/3 and V/4 or cirri V/2 and VI/2. Transverse cirri subterminal. 11–16 right and 12–19 left marginal cirri. Four dorsal kineties. Three narrowly spaced caudal cirri cilia of which are distinctly long.

**Table 1:** Morphometric characterization of *Quadristicha subtropica* n. sp.

Character^a^	HT	Min	Max	Med	Mean	SD	CV	*n*
Body length	119	81	120	94.9	97.3	9.4	9.7	25
Body width	54	30	55	40.1	40.1	5.1	12.6	25
Body length to width, ratio	2.20	2.17	2.99	2.37	2.44	0.21	8.67	25
Adoral zone, length	36	27	36	30.4	30.2	1.9	6.4	25
Adoral zone length to body length, ratio	0.30	0.27	0.37	0.31	0.31	0.03	8.7	25
Number of adoral membranelles	20	19	21	20	19.8	0.5	2.6	25
Number of BC	1	1	1	1	1.0	0	0	25
Number of FC	3	3	3	3	3.0	0	0	25
Number of FVC	4	4	4	4	4.0	0	0	25
Number of PVC	3	3	3	3	3.0	0	0	25
Number of PTVC	2	2	2	2	2.0	0	0	25
Number of TC	5	5	5	5	5.0	0	0	25
Number of cirri in LMR	19	12	19	14	14.7	1.7	11.6	25
Number of cirri in RMR	13	11	16	13	13.2	1.3	10.1	25
Number of CC	3	3	3	3	3.0	0	0	25
Number of DK	4	4	4	4	4.0	0	0	25
Number of bristles in DK1	11	8	12	10	9.9	1.0	10.0	22
Number of bristles in DK2	12	11	14	12	12.2	0.9	7.0	22
Number of bristles in DK3	13	12	14	12	12.5	0.7	5.4	22
Number of bristles in DK4	7	5	7	6	6.4	0.6	9.2	22
Total number of dorsal cilia	43	37	45	41	40.9	2.2	5.3	22
Distance between cirrus III/2 and undulating membranes	2	1	4	1.5	1.6	0.6	36.2	25
Distance between cirri V/2 and V/3	16	6	16	12.1	11.2	3.4	30.3	23
Distance between cirri V/2 and VI/2	12	7	15	10.9	10.6	1.8	17.4	25
Distance between cirri V/3 and V/4	14	9	16	10.5	10.8	1.7	15.5	25
Distance between cirrus II/1 and anterior end of body	81	55	75	64.3	65.3	5.9	9.0	25
Distance between cirrus IV/3 and anterior end of body	35	26	35	28.2	28.9	2.1	7.3	25
DE value	0.19	0.13	0.25	0.20	0.19	0.03	14.7	25
Number of Ma	2	2	2	2	2.0	0	0	25
Number of Mi	1	1	1	1	1.0	0	0	25
Macronuclear nodule, length	21	10	25	14.1	15.2	4.1	27.0	25
Macronuclear nodule, width	12	6	12	7.3	7.9	1.7	21.9	25
Micronucleus, diameter	4	3	5	3.1	3.4	0.5	16.1	25

a All data are based on protargol-stained specimens; measurements in μm.

CV, coefficient of variation in %; HT, holotype; Max, maximum; Mean, arithmetic mean; Med, median; Min, minimum; *n*, sample size; no., number; and *SD*, standard deviation.

#### Type Material

The protargol slide (registry no. GQ2018091201A) with the holotype specimen (Figures 1E,F, 2F) and four paratype slides (registry no. GQ2018091201B–E) were deposited in the Laboratory of Protozoological Biodiversity and Evolution in Wetland, Shaanxi Normal University, China. A paratype slide (registry no. GQ2018091201F) with protargol-stained specimens is deposited in the Laboratory of Protozoology, Ocean University of China.

#### Type Locality

A freshwater pond in Peninsula Lake Park (18°41′03″N; 110°24′02″E) in Wanning, China.

#### Etymology

The Latin adjective *subtropicus*, *−a, −um* (masc., fem., neut.) recalls the fact that the type material was found in a subtropical area of China.

### Morphological Description

Cells in interphase *in vivo* 60–115 μm × 25–45 μm (*n* = 12), and after protargol staining 81–120 μm × 30–55 μm. Ratio of length to width after protargol staining about 2.4:1 (Figures 1A–F, 2A–K and Table 1). Body ellipsoid or elongate ovoid, flexible, but not contractile (Figures 1A,B, 2A–C). Two closely spaced ellipsoidal macronuclear nodules (Ma) about 10–25 μm × 6–12 μm in size (after protargol staining), located in mid-body region slightly left of midline. One globular micronucleus (Mi), about 3 μm in diameter (after protargol staining), located between macronuclear nodules (Figures 1F, 2K). Contractile vacuole located ahead of mid-region of body near left margin, about 13 μm across, contracting at intervals of about 12 s (Figures 1A,B, 2C). Cortical granules absent. Cytoplasm grayish, containing a mass of irregular crystals (about 2–10 μm large, and many lipid droplets (1–2 μm dia.), which render cell opaque and dark at low magnifications (Figure 2E). Movement moderately rapid gliding. Resting cyst spherical with smooth surface, about 40 μm in diameter (Figures 1C, 2D).

Infraciliature as shown in Figures 1E,F, 2F,G. Adoral zone about 31% of cell length, 27–36 μm long in protargol preparations, composed of 19–21 (*n* = 25) membranelles. DE-value ca. 0.2 (*n =* 25). Paroral (P) and endoral (E) short, intersecting at middle region, and inconspicuously curved (Figures 1D,H and Table 1).

Eighteen frontal-ventral-transverse cirri: three slightly enlarged frontal cirri (FC) near distal portion of adoral zone of membranelles (AZM), cilia about 19 μm long *in vivo*; buccal cirrus (BC) near anterior end of paroral; four frontoventral cirri, cirrus III/2 slightly ahead of level of cirrus VI/3, cirrus IV/3 at about level of buccal vertex; three postoral ventral cirri located behind buccal vertex, with cirrus IV/2 arranged anterior to level of cirrus V/4, distance between cirri V/3 and V/4 slightly shorter than that between cirri V/3 and V/2; two pretransverse ventral cirri, cirrus VI/2 located between the levels of cirri II/1 and III/1, distance between cirri V/2 and VI/2 slightly shorter than that between cirri V/2 and V/3; five transverse cirri (TC) located about three-quarters down length of body, bases distinctly enlarged, cilia about 23 μm long *in vivo* and slightly protruding beyond posterior cell margin (Figures 1D,E, 2F,H,I). Marginal cirri are disposed in two rows, on the right and left of the cell, respectively, composed of 11–16 and 12–19 cirri, respectively, in life about 16 μm long; left marginal row commences at level of buccal vertex and terminates subcaudally, while right marginal row (RMR) commences slightly below level of buccal vertex (Figures 1E, 2F).

Invariably in four dorsal kineties (DK) with about 5 μm long cilia composed of 8–12, 11–14, 12–14, and 5–7 dikinetids, respectively. Dorsal kineties 1–3 almost bipolar with kinety 1 always with a wide gap in posterior portion. Dorsal kinety 4 terminates at about mid-body. Three narrowly spaced caudal cirri (CC) located at posterior body margin, one each at posterior end of dorsal kineties 1–3; cilia of caudal cirri conspicuously long, about 25 μm *in vivo* (Figures 1F, 2G,J).

### Divisional Morphogenesis

#### Stomatogenesis

Opisthe: The earliest cortical morphogenetic event is the apokinetal appearance of a small patch of basal bodies (kinetosomes) in irregular arrangement, the oral primordium (OP; Figures 3A–I, 4A–D, 5A–M). Subsequently, a long and narrow oral primordium is formed (Figures 3B,C, 5B,C). The membranelles of the opisthe’s adoral zone organize in a posterior direction. Simultaneously, the anlage for the undulating membranes (UM-anlage) is formed to the right of the oral primordium as a streak of basal bodies (Figures 3D,E, 5D,E). Later, the membranelles of the opisthe’s adoral zone are organized completed and the anterior end of the newly built adoral zone bends to the right, forming the new oral structure. It is suggested that the leftmost frontal cirrus is generated from the anterior end of the undulating membrane-anlage (= anlage I). Subsequently, the undulating membrane-anlage of both the proter and the opisthe is separated from which the endoral and paroral are formed (Figures 3H, 4A,C, 5H).

Proter: The parental AZM is retained by the proter, so changes to the oral structure are confined to the paroral membranes and endoral membranes. The UM-anlage is formed by the dedifferentiation of the parental undulating membranes. In subsequent stages, the basic development of the UM-anlage follows a similar pattern to that in the opisthe (Figures 3F,H, 4A,C, 5F,H,J,M).

#### Development of Cortical Ciliature

Along with the organization of the membranelles of the opisthe’s adoral zone, division continues with the formation of the development of the frontoventral-transverse cirral anlagen (FVT-anlagen). We failed to obtain specimens in the stage between those as shown in Figures 3E,F and hence were unable to determine the origin of anlagen II to VI. We speculate that FVT-anlagen I and II in the opisthe develop *de novo*, and cirri IV/3, IV/2, and V/4 contribute the formation of the FVT-anlagen. Five thread-like anlagen are formed in both proter and opisthe (Figures 3F, 5F). Subsequently, cortical morphogenesis proceeds with the cirral segregation from these streaks. After migration and differentiation, 17 cirri will be formed from each group, whereas the remaining one (the leftmost frontal cirrus) may derive from the undulating membrane-anlage (Figures 3H, 4A,C, 5H,J,M). Finally, the constant 18 cirri are formed within the anlagen I–VI as follows: 1, 3, 3, 3, 4, and 4 cirri.

The anteriormost marginal cirri and some cirri near the prospective division furrow of the marginal rows disaggregate to form the marginal anlagen [left marginal anlagen (LMA) and right marginal anlagen (RMA); Figures 3F, 5F]. The new marginal cirri then develop and replace the old ones (Figures 3H, 4A,C, 5H,J,M).

New dorsal kineties are formed in a typical *Urosomoida* pattern. Firstly, within dorsal kineties 1, 2, and 3, basal bodies are proliferated to form dorsal kineties anlagen (DKA) at two sites above and below the prospective division furrow (Figures 3G,I, 4B,D, 5G). In the later stage, a gap is always present in posterior portion of kinety 1 (Figure 4D). Subsequently, above the anteriormost portion of the proter’s and opisthe’s right marginal primordia a short streak of paired basal bodies develops, viz. the anlage for the shortened dorsal kinety 4 (Figures 3H, 5H). The posterior ends of the new dorsal kineties 1, 2, and 3 commence with the differentiation of caudal cirri (Figures 3I, 4B,D, 5L).

#### Division of Nuclear Apparatus

The nuclear apparatus divides in the usual way and hence requires no further comment (Figures 3G,I, 4B,D, 5I,K).

### Phylogenetic Analyses Based on SSU rDNA Gene Sequences

The SSU rDNA sequence of *Quadristicha subtropica* n. sp. was deposited in GenBank with the accession number MZ338339. The length and GC content of the new sequence are 1,627 bp and 45.09%, respectively.

**Figure 6:**
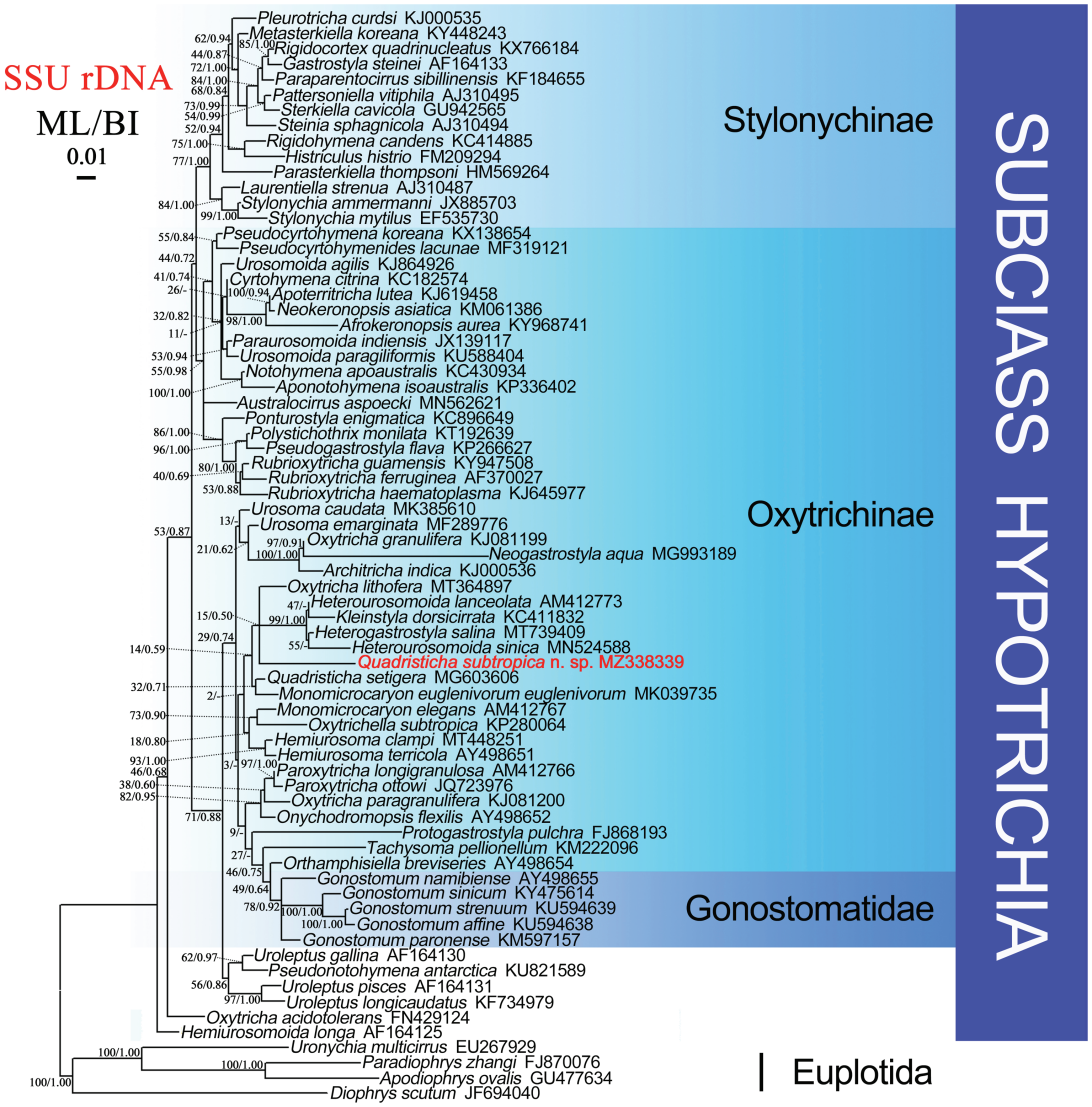
Maximum likelihood (ML) tree based on SSU rDNA sequence data. *Quadristicha subtropica* n. sp. is indicated in bold. Support values for nodes are for ML and BI (ML/BI), respectively. Clades with a different topology in the BI tree are indicated by “-”. All branches are drawn to scale. The scale bar corresponds to 1 substitution per 100 nucleotide positions.

Phylogenetic trees using two different methods (ML and BI) had almost identical topologies; therefore, only the ML tree is presented with support values from both algorithms at the nodes (Figure 6). *Quadristicha subtropica* n. sp. nests within a poorly supported clade (ML/BI, 15/0.50) that also contains *Heterogastrostyla salina* Lu et al., 2020, *Heterourosomoida lanceolata* (Berger, 1999) Singh and Kamra, 2014, *Heterourosomoida sinica* Wang et al., 2020, *Kleinstyla dorsicirrata* Singh and Kamra, 2014, and *Oxytricha lithofera* Foissner, 2016. *Quadristicha setigera,* the only congener of *Q. subtropica* n. sp., clusters with *Monomicrocaryon euglenivorum euglenivorum* (Kahl, 1932) Foissner, 2016 with low support (ML/BI, 32/0.71). The two clades containing species of *Quadristicha* are sister groups. Given the low support values across the tree, the present phylogeny is far from robust despite the fact that several preliminary phylogenetic analyses were performed using different taxon samples and outgroup species.

The similarities of the SSU rDNA sequence of *Q. subtropica* n. sp. to *H. sinica*, *H. lanceolata*, *K. dorsicirrata*, *O. lithofera, H. salina,* and *Q. setigera* are 89.3, 90.3, 90.3, 90.7, 93.8, and 93.8%, respectively.

## Discussion

### Comparison With Closely Related Species

*Quadristicha subtropica* n. sp. differs from the type species *Q. setigera* in its body size *in vivo* (60–115 μm × 25–45 μm vs. 40–60 μm × 15–21 μm), length of dorsal cilia (about 5 μm vs. 10–15 μm), number of adoral membranelles (19–21 vs. 13–18), number of right (11–16 vs. 3–8) and left (12–19 vs. 6–8) marginal cirri, location of the anterior termination of the RMR (ahead of the level of the buccal vertex vs. behind the level of the buccal vertex), endoral and paroral slightly (vs. strongly) curved, location of the buccal cirrus near the anterior (vs. posterior) end of the paroral, and location of cirrus IV/3 at about (vs. behind) the level of the buccal vertex (Berger, 1999; Kim et al., 2020).

In terms of the features, such as (1) two macronuclear nodules with a micronucleus in between, (2) four dorsal kineties without fragmentation of kinety 3, (3) cortical granules absent, and (4) three prolonged caudal cirri, *Q. subtropica* n. sp. should be compared with six *Monomicrocaryon* species, i.e., *Monomicrocaryon alfredi* (Berger, 1999) Foissner, 2016, *Monomicrocaryon crassistilata* (Kahl, 1932) Foissner, 2016, *Monomicrocaryon halophilum* (Kahl, 1932) Foissner, 2016, *Monomicrocaryon kahlovatum* (Berger, 1999) Foissner, 2016, *Monomicrocaryon parahalophilum* (Wang and Nie, 1935) Foissner, 2016, and *Monomicrocaryon sphagni* (Kahl, 1932) Foissner, 2016.

*Quadristicha subtropica* n. sp. can be separated from *M. alfredi* by the RMR starting below the level of cirrus VI/3 (vs. ahead of the level of cirrus VI/4), and cilia of caudal cirri protruding rightward (vs. straight or indistinct) (Berger, 1999; Foissner, 2016).

*Quadristicha subtropica* n. sp. can be distinguished from *M. halophilum* by the locations of the rightmost frontal cirrus slightly (vs. distinctly) ahead of the level of the buccal cirrus, transverse cirri (subcaudal vs. caudal), cirrus IV/2 (ahead of vs. below) cirrus V/4 and the anterior termination of the RMR (below the level of cirrus VI/3 vs. ahead of the level of cirrus VI/4), and also the number of dikinetids in dorsal kinety 1 (8–12 vs. 18 or 21 in population from Kahl, 1932, data from drawings), cilia of caudal cirri protruding rightward (vs. straight) and the habitat (fresh water vs. saline water) (Berger, 1999; Foissner, 2016).

*Quadristicha subtropica* n. sp. can be separated from *M. parahalophilum* by the location of the transverse cirri (subterminal vs. terminal), and the number of dikinetids in dorsal kinety 1 (8–12 vs. 27 in population from Wang and Nie, 1935, data from drawing) (Wang and Nie, 1935; Berger, 1999; Foissner, 2016).

*Monomicrocaryon crassistilata* resembles *Q. subtropica* n. sp. reasonably well; however, the former differs from the latter in the length of the dorsal cilia (8–10 μm vs. 5 μm) and the orientation of the caudal cirri (straight vs. projecting rightward) (Berger, 1999; Foissner, 2016).

*Quadristicha subtropica* n. sp. can be separated from *M. sphagni* by the length of the dorsal cilia (5 μm vs. about 15 μm), the ratio of body length to width *in vivo* (about 3:1 vs. about 5:1, data from drawing), the location of the transverse cirri (subcaudal vs. caudal), and the location of cirrus IV/3 (at about the level of the buccal vertex vs. ahead of the level of the buccal vertex) (Berger, 1999; Foissner, 2016).

*Monomicrocaryon kahlovatum* can be separated from *Q. subtropica* n. sp. by its oval (vs. ellipsoid or elongate ovoid) body shape and the orientation of the caudal cirri (straight vs. protruding rightward) (Berger, 1999; Foissner, 2016).

### Morphogenetic Comparison

Until now, the cortical morphogenesis of *Q. subtropica* n. sp. is the only detailed study within the genus *Quadristicha*, which proceeds in a similar way to other members of the family Oxytrichidea (Berger, 1999).

Berger (1999) documented some middle and late stages of morphogenesis in *Q. setigera*. Based on these and the present data, the mid-to-late stages of the two congeners are consistent (Berger, 1999). The early stages of morphogenesis in *Quadristicha* are revealed here for the first time.

### Phylogenetic Analyses

The genus *Quadristicha* comprises two species, namely, *Q. setigera* (type species) and *Q. subtropica* n. sp. both of which were included in the present phylogenetic analyses. *Quadristicha subtropica* n. sp. groups with *H. salina*, *H. lanceolata*, *H. sinica*, and *K. dorsicirrata*. The close relationship among these species is supported by several morphological features including: a flexible pellicle, two macronuclear nodules, one marginal cirral row on each side, two pretransverse cirri and five transverse cirri, and four dorsal kineties (Berger, 1999; Singh and Kamra, 2014; Foissner, 2016; Lu et al., 2020; Wang et al., 2021b).

## Data Availability Statement

The datasets presented in this study can be found in online repositories. The names of the repository/repositories and accession number(s) can be found at https://www.ncbi.nlm.nih.gov/genbank/, MZ338339.

## Author Contributions

CS and QG collected the samples and carried out almost all of the experiments (preparations, illustrations, micrographs, etc.). AW was responsible for the language correction. JW did the identification of the species and revised the manuscript. All authors contributed to the article and approved the submitted version.

### Conflict of Interest

The authors declare that the research was conducted in the absence of any commercial or financial relationships that could be construed as a potential conflict of interest.
